# Seasonal changes in the preen wax composition of the Herring gull *Larus argentatus*

**DOI:** 10.1007/s00049-017-0239-z

**Published:** 2017-07-19

**Authors:** Izabela Fischer, Łukasz P. Haliński, Włodzimierz Meissner, Piotr Stepnowski, Małgorzata Knitter

**Affiliations:** 1 Avian Ecophysiology Unit, Department of Vertebrate Ecology and Zoology, University of Gdańsk, Wita Stwosza 59, 80-308 Gdańsk, Poland; 2 Department of Environmental Analysis, Faculty of Chemistry, University of Gdańsk, Wita Stwosza 63, 80-308 Gdańsk, Poland

**Keywords:** Herring gull, *Larus argentatus*, Preen waxes, Gas chromatography–mass spectrometry

## Abstract

The preen gland produces oily secretion, which smeared onto a bird’s plumage improves its maintenance. The main components of the secretion are waxes, and its composition often changes during the year. The aim of this study was to determine the differences in the chemical composition of preen waxes in adult herring gulls *Larus argentatus,* captured in Poland in winter and in the breeding season. Preen gland secretions of herring gulls consist of monoester waxes, composed of about 29 saturated C_7_–C_16_ fatty acids and about 51 saturated C_11_–C_20_ alcohols. Unbranched-octanoic acid and *n*-hexadecanol dominated fatty acid and alcohol fractions, respectively, but 2-methyl-branched compounds were numerous in all individuals. The chemical compositions of fatty acids and alcohols differ between winter and the breeding season. In breeding gulls, 2-monomethyl-branched fatty acids were lower in content or could not be found, contrary to herring gulls in winter, where 2-monomethyl-substituted fatty acids were the second most abundant among all the fatty acids. Breeding gulls had also a higher content of *n*-octanoic acid and *n*-hexadecanol and a lower content of 2,6- and 2,8-dimethyl-substituted fatty acids than individuals caught during the winter. Differences in fatty acid composition were greater in breeding males, which incubate more often at night than breeding females. Hence, chemical changes in the preen wax composition in males may have evolved as additional nocturnal protection against mammalian predators which use olfaction to detect their prey and which are more active at night; however, this needs to be tested. Olfactory-based mate recognition in the colony also cannot be excluded at this stage of experimentation.

## Introduction

The preen gland is the only skin gland that exists in most birds (Kardong 2014). Its secretion contains mainly waxes, i.e., esters of fatty acids and long-chain alcohols, forming a mixture of dozens of compounds which differ in chemical structure, like carbon chain length or the location of branches. The composition of preen waxes is thought to be characteristic for certain species, but some intraspecific changes may appear among birds depending on the season (Kolattukudy et al. 1985, 1987; Reneerkens et al. 2006), age (Kolattukudy and Sawaya 1974), diet or captivity (Thomas et al. 2010).

The chemical composition of preen gland secretion often changes during the breeding season, which decreases the risk of the predation of the incubating birds by reducing the bird’s smell (Reneerkens et al. 2005), producing a repulsive smell (Martín-Vivaldi et al. 2010; Röder et al. 2014) or producing a smell that can possibly help birds blend into the environment (Soini et al. 2007). The first mechanism has been observed in mallards (*Anas platyrhynchos*) and red knots (*Calidris canutus*), which produce diester waxes during courtship and incubation, and monoesters during the rest of the year (Kolattukudy et al. 1987; Piersma et al. 1999; Reneerkens et al. 2006). Diesters produced during the breeding season have a higher molecular weight, thus they are less volatile and more difficult to be detected by mammalian predators which use olfaction to detect their prey (Reneerkens et al. 2005). These changes in the chemical composition of preen gland secretion in the breeding season are observed only in the sex that incubates (Reneerkens et al. 2007a).

Two other types of changes observed in breeding individuals affect volatile compounds that occur in preen gland secretion. The uropygial gland secretion of breeding hoopoes (*Upupa epops*) produces a very strong repulsive smell that may repel predators (Martín-Vivaldi et al. 2010). In contrast, in the junco (*Junco hyemalis*) a much higher content of volatile alcohols was found in the preen gland secretion of breeding individuals (Soini et al. 2007). The junco builds its nest on the ground surrounded by plants containing alcohols on the surface of their leaves, and it has been suggested that these changes in preen gland secretion may diminish the risk of being detected by mammalian predators. Recent studies on preen oil have suggested that changes in its chemical composition during the breeding season could facilitate individual recognition or mate choice (Campagna et al. 2012; Caro et al. 2015).

The aim of this study is to determine differences in the chemical composition of preen gland secretion in males and females of adult herring gulls (*Larus argentatus*) captured in winter and in the breeding season. This species breeds in colonies, which clearly affects an animal’s individual fitness due to both the costly and beneficial consequences of the close proximity of conspecifics. Gull colonies are inherently conspicuous due to the permanent presence of multiple residents, sound and smell, which may increase predation risk. On the other hand, incoming predators may be more likely to be detected and more effectively deterred (review in Brown and Brown 2001). Hence, colonial breeding might have an influence on the chemical composition of preen gland secretion by diminishing both the seasonal and sex-related differences which were found in related species nesting solitarily (Kolattukudy et al. 1987; Reneerkens et al. 2006).

In this study we were interested also in investigating the preen wax composition of the herring gull, as the chemical composition of preen waxes in gulls has barely been discussed in the literature. Zeman and Jacob (1972) presented details on the composition of fatty acids and alcohols in preen waxes in three gull species, including the herring gull, but with no information about the bird’s age, sex or the season. Recently, Leclaire et al. (2012) studied intact waxes collected in the pre-laying period of black-legged kittiwakes (*Rissa tridactyla*), i.e., a species related to herring gulls that also belongs to the Laridae family, but their study was aimed at the possible function of body odour for mate recognition.

## Materials and methods

### Bird capturing and measuring

Herring gulls were captured during winter from December to February 2010/2011, and during spring from late April to mid-May in both 2011 (2 individuals) and 2012 (15 individuals). In total, 34 adult herring gulls were captured, including 17 individuals (nine males and eight females) in winter from the rubbish dump in Gdańsk Szadółki (54°19′N, 18°32′E) and 17 individuals (nine males and eight females) during spring, in the breeding season while incubating eggs, from breeding colonies in Ustka (54°35′N 16°51′E) and Łeba (54°45′N 17°33′E), northern Poland. The birds were aged according to plumage characteristics (Malling Olsen and Larsson 2004) and only birds classified as adult, i.e., being at least in the fifth calendar year of life, were used in this study. Birds in winter were captured using fishing line loop (Meissner 2010) and birds in the breeding season were captured with cage nest traps (Bub 1968). Each bird was weighed with the accuracy of 1 g, with an electronic balance, and standard measurements were taken: total head length and bill depth at the gonys with 0.1 mm accuracy, to be used as discrimination functions to determine bird sex, because these two measurements differ between sexes to the greatest extent (Bosch 1996). The body masses of the birds used in this study were in the range of those given in the literature (Cramp and Simmons 1983). Thus, it was assumed that the samples of preen waxes were obtained from herring gulls that were in good condition.

### Bird sexing

Gulls are monomorphic with respect to plumage characteristics (Burger and Gochfeld 1996). To determine the sex of caught individuals about 100 μl of blood was collected from the brachial vein and used for molecular analysis based on the identification of fragments of two chromo-helicase-DNA-binding genes (CHD-Z and CHD-W), which occur on sex chromosomes in birds (Griffiths et al. 1998). DNA was isolated from blood samples using a Blood Mini Kit (A&A Biotechnology). PCR was performed on a Mastercycler (Eppendorf). Seventeen individuals captured during winter were sexed using 1237L/1272H primers (Kahn et al. 1998). Molecular sexing of seven wintering individuals was, however, ineffective. These individuals were re-analyzed using 2550F/2718R primers (Fridolfsson and Ellegren 1999), but in five individuals it was still ineffective. The sex of these birds was determined by discrimination functions based on total head length and bill depth (Meissner et al. 2017). Seventeen individuals captured in the breeding season were sexed using 2550F/2718R primers.

### Sample collection

Preen gland secretion was collected by swabbing the top of the preen gland papilla with a cotton pad. Swabs were placed individually in glass tubes with PTFE-sealed caps (Duran) and stored at 4 °C. Each swab was extracted with 5 ml of ethyl acetate in a 15 min ultrasonic bath at room temperature.

### Chemical analysis

The chemical analysis of preen waxes was performed using three chromatographic techniques: (1) thin-layer chromatography (TLC) to identify the main classes of compounds that occur in intact preen gland secretion, (2) gas chromatography with a flame-ionization detector (GC–FID), performed after the hydrolysis of preen waxes to respective fatty acids and alcohols, to determine the relative composition of each fraction and (3) gas chromatography coupled to mass spectrometry (GC–MS) to identify the fatty acid and alcohol moieties of preen waxes.

The main classes of compounds in preen gland secretion were identified using thin-layer chromatography TLC. Samples were developed on classical silica gel 60 plastic plates 20 × 20 cm (Merck) with a mixture of hexane–ethyl ether-formic acid (v/v 90:10:1) as the developing solvent (Kolattukudy et al. 1985) and beeswax as the reference standard (in a way similar to Thomas et al. 2010). Compounds were visualised with 10% 2′,7′-dichlorofluorescein solution in propanol (Fluka) under UV light.

For a detailed study of their chemical composition, preen waxes were hydrolyzed to fatty acid and alcohol moieties. An aliquot of ca. 400 μl of each extract was taken and ethyl acetate was removed under a gentle stream of nitrogen. Preen waxes were saponified by adding 100 μl of a 0.5 M solution of KOH in 95% ethanol and heating the mixture at 70 °C for 3 h. The alcohol fraction was then separated by extraction with 2 ml *n*-hexane, while fatty acids remained present as their sodium salts in the ethanol–water phase. Solvents were evaporated from both fractions under the stream of nitrogen (at 70 °C in the case of the fatty acid fraction) and samples were then kept at 4 °C. Just before the analysis, 100 μl BSTFA with TMCS (99:1; Sigma-Aldrich) was added to the samples and the fatty acids and alcohols were transferred to trimethylsilyl (TMSi) esters and trimethylsilyl ethers, respectively (30 min at 90 °C).

Each sample was analyzed using gas chromatography with a flame-ionization detector (GC–FID) performed on a Trace 2000 Series GC (ThermoQuest CE Instruments). Separation was carried out on a PE-1 column (Perkin Elmer, 30 m × 0.32 mm, film thickness of the stationary phase 0.25 μm), under conditions as follows: injector temperature 310 °C, oven temperature from 60 to 270 °C at 5 °C min^−1^, FID temperature 310 °C. Argon was used as the carrier gas at a flow rate of 1 ml min^−1^, and the total time of analysis was 42 min.

Fatty acids (as TMSi esters) and alcohols (as TMSi ethers) liberated from preen waxes were identified using gas chromatography coupled to mass spectrometry (GC–MS). The analysis of selected samples was carried out using GCMS-QP2010SE (Shimadzu Corporation) with a Rtx-5MS column (Restek, 30 m × 0.25 mm, film thickness of the stationary phase 0.25 μm) with helium as the carrier gas at a flow rate of 1 ml min^−1^. The injection temperature was set at 310 °C, the oven temperature was programmed from 60 to 260 °C at 5 °C min^−1^, the MS ion source temperature was 200 °C and the interface temperature was 310 °C. The total run time was 40 min. Electron ionisation (EI) mass spectra were recorded in the full-scan mode at the electron energy of 70 eV.

Fatty acids (as TMSi esters) and alcohols (as TMSi ethers) were identified by comparing their mass spectra with the NIST 08 mass spectra library and with the literature (Jacob 1975, 1976; Tulloch 1985; Rontani and Aubert 2004). Additionally, patterns of fragment ions were determined for 2-methyl-branched fatty acids and 2-methyl-branched alcohols by analysing standards of 2-methylhexadecanoic acid and 2-methyl-1-hexadecanol (synthesised by the Jagiellońskie Centrum Innowacji, Kraków, Poland) in conditions as described above. Some of the compounds bearing more than one methyl branch in the molecule were not fully characterized.

The relative compositions of fatty acid and alcohol fractions were calculated based on the peak area of each compound in relation to the total area of all peaks in the GC–FID analysis of that certain fraction.

### Comparison of preen wax composition

Due to a high number of compounds detected in each fraction, only those that occurred in all individuals and which had an average abundance above 1% in at least one season were chosen for the comparison analysis. Compounds with a mean abundance less than 1% were considered insignificant.

### Statistical analyses

For statistical analysis, the data were transformed using the arcsine square root transformation (Zar 1996). For the reduction of variables, principal component analysis (PCA) was applied. Principal components explaining together over 80% of the total variance were selected (Stanisz 2007) and only factor loadings with an absolute value above 0.7 were considered significant (Grabiński 1992). Differences in the abundance of fatty acids and alcohols were tested using MANOVA. All statistical analyses were performed using Statistica 10 software (StatSoft).

## Results

### Composition of preen gland secretion

**Fig. 1:**
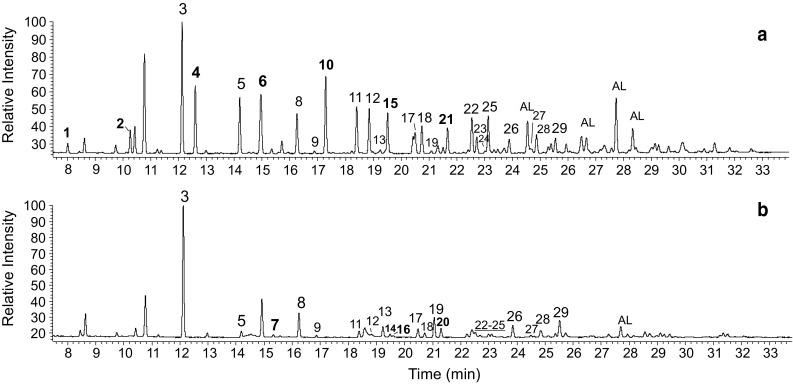
Representative gas chromatograms of the fatty acid fraction (as TMSi esters) of preen waxes in adult herring gulls in **a** winter and **b** the breeding season. *Numbered* peaks represent fatty acids as given in Table 1. Fatty acids detected only in some individuals are marked in *bold*. A relatively simple extraction method resulted in the presence of alcohols (marked as ‘AL’) in some of the fatty acid fractions

**Fig. 2:**
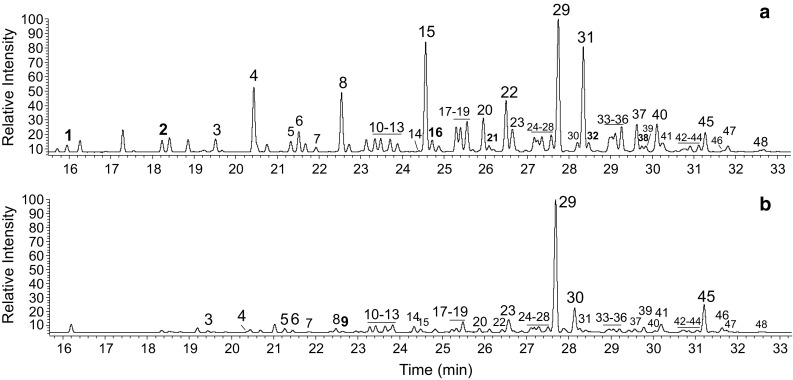
Representative gas chromatograms of the alcohol fraction (as TMSi ethers) of preen waxes in adult herring gulls in **a** winter and **b** the breeding season. *Numbered* peaks represent alcohols as given in Table 2 and the *horizontal line* shows the area where peaks *x*–*y* (numbered from *x* to *y*) were present. Alcohols detected only in some individuals are marked in *bold*

The TLC analysis of intact preen gland secretion showed that the main components of preen gland secretion in adult herring gulls were monoester waxes. About 29 saturated C_7_–C_16_ fatty acids (i.e., varying from 7 to 16 of the total number of carbon atoms in the molecule; Fig. 1; Table 1) and about 51 of saturated C_11_–C_20_ alcohols (Fig. 2; Table 2) were detected.

**Table 1:** The relative composition of the fatty acid fraction of preen waxes in adult herring gulls during winter and during the breeding season

Peak no.	Total carbon atom no.	Branch^a^	Winter				Breeding season			
			Range		Mean (%)	SD (%)	Range		Mean (%)	SD (%)
			Min (%)	Max (%)			Min (%)	Max (%)		
1	7	2-m	0.2	1.4	0.84	0.30				
2	8	2-m	0.7	3.3	2.50	0.58	0.0	1.7	0.40	0.55
3	8	*n*	12.0	29.8	17.13	4.23	21.9	48.2	36.92	8.41
4	9	2-m	1.9	11.7	8.10	2.31	0.0	5.6	1.32	1.82
5	10	2,6-m	4.7	10.4	7.80	1.28	0.4	6.9	3.22	1.97
6	10	2-m	+				±			
7	11	?					0.0	0.6	0.26	0.26
8	11	2,6-m	4.2	10.6	6.46	1.68	1.1	11.4	5.97	2.80
9	10	*n*	0.2	0.5	0.34	0.08	0.3	0.9	0.66	0.20
10	11	2-m	3.8	13.1	9.00	2.43	0.0	7.4	1.96	2.62
11	12	2,6-m	6.0	7.5	6.71	0.43	0.6	6.4	3.61	1.90
12	12	2,8-m	4.4	6.3	5.80	0.44	0.3	5.8	2.42	1.88
13	13	?	0.3	1.0	0.57	0.18	0.5	4.2	2.28	1.43
14	13	?					0.0	1.8	0.62	0.64
15	12	2-m	3.6	5.6	4.78	0.60	0.0	4.6	1.56	1.85
16	13	?					0.0	0.6	0.23	0.24
17	13	2,6-m	2.1	4.4	3.04	0.60	1.1	4.4	3.12	0.85
18	13	2,8-m	2.7	4.9	3.83	0.63	0.8	4.6	3.04	1.30
19	14	2,4,8-m	0.2	1.0	0.48	0.21	0.3	8.0	3.69	2.89
20	14	?					0.0	6.6	1.66	1.87
21	13	2-m	1.1	4.8	3.02	1.19	0.0	5.9	1.56	1.93
22	14	2,6-m	3.6	6.6	5.19	0.66	1.6	8.3	4.04	1.98
23	14	2,8-m	1.4	2.7	2.03	0.35	0.6	3.7	1.88	0.99
24	15	?	0.1	0.4	0.26	0.07	0.3	3.0	1.05	0.71
25	14	?	3.7	5.8	4.81	0.47	0.9	5.7	3.04	1.69
26	15	2,6,10-m	1.4	2.7	2.07	0.40	2.1	7.0	4.66	1.57
27	15	2,8	0.3	0.8	0.43	0.14	0.3	1.6	0.87	0.40
28	15	?	1.8	4.3	2.43	0.54	2.5	7.7	4.69	1.51
29	16	2,6,?-m	1.4	2.9	2.09	0.46	1.7	9.6	5.27	2.49

Compound numbers correspond to the peak numbering in Fig. 1

^a^Location of methyl groups in the chain; *n*-unbranched, ?*-*unidentified, ‘*+*’-present

**Table 2:** The relative composition of the alcohol fraction of preen waxes in adult herring gulls during winter and during the breeding season

Peak no.	Total carbon atom number^a^	Branch^b^	Winter				Breeding season			
			Range		Mean (%)	SD (%)	Range		Mean (%)	SD (%)
			Min (%)	Max (%)			Min (%)	Max (%)		
1	11	2-m	0.1	0.6	0.28	0.14				
2	12	2-m	0.2	1.8	0.75	0.36				
3	13	2,?	0.4	1.4	0.88	0.35	>0.0	0.7	0.18	0.23
4	13	2-m	1.9	5.8	4.42	1.03	>0.0	3.7	1.05	1.18
5	14	2,?	0.6	1.6	0.95	0.24	0.4	1.3	0.80	0.23
6	14	2,?	1.2	2.0	1.66	0.24	0.1	1.6	0.79	0.45
7	14	2,?	0.2	0.5	0.38	0.09	>0.0	0.3	0.12	0.13
8	14	2-m	3.1	5.4	4.79	0.61	0.8	4.7	2.51	1.35
9	14	?					>0.0	0.6	0.23	0.24
10	15	2,?	0.9	1.8	1.38	0.33	0.8	1.9	1.32	0.34
11	15	2,?	0.9	1.6	1.29	0.24	0.8	2.1	1.42	0.37
12	15	2,?	0.8	1.7	1.33	0.30	0.4	2.0	1.18	0.43
13	14	?	0.5	1.0	0.75	0.15	0.8	2.8	1.70	0.47
14	16	?	>0.0	0.3	0.13	0.06	0.1	1.7	0.62	0.53
15	15	2-m	4.9	11.6	8.35	1.70	1.0	9.3	3.85	2.70
16	15	2,?	0.2	3.0	1.22	0.71				
17	16	2,?	1.6	2.4	2.12	0.16	0.8	2.1	1.42	0.37
18	16	2,?	1.8	2.6	2.15	0.23	1.1	2.2	1.69	0.39
19	16	2,?	2.4	3.3	3.01	0.23	2.2	4.5	2.85	0.54
20	16	2,?	2.4	3.2	2.91	0.22	1.2	3.6	2.42	0.82
21	16 + 17	? + ?	0.1	1.5	0.60	0.36				
22	16	2-m	3.8	5.1	4.51	0.38	0.9	4.5	2.73	1.32
23	16 + 17	? + ?	1.9	4.9	2.88	0.74	2.0	5.8	3.76	1.08
24	17	2,?	1.1	2.0	1.37	0.24	1.0	1.8	1.41	0.21
25	17	2,?	0.8	1.4	1.03	0.17	0.9	1.6	1.26	0.21
26	17	2,?	1.3	2.3	1.68	0.31	1.1	1.9	1.60	0.23
27	17	?	>0.0	0.4	0.12	0.10	>0.0	0.3	0.09	0.10
28	17	?	1.3	2.1	1.75	0.23	1.5	2.9	2.19	0.46
29	16	*n*	12.7	25.8	17.28	3.49	22.3	40.9	29.82	6.00
30	18	?	0.6	2.4	1.20	0.46	0.8	7.8	4.14	2.41
31	17	2-m	5.1	11.4	7.93	1.71	1.1	9.8	4.16	2.72
32	17	2,?	0.4	2.8	0.99	0.61				
33	18	?	0.8	1.2	1.01	0.10	0.4	1.2	0.83	0.27
34	18	2,?	0.7	1.4	0.96	0.19	0.6	1.8	1.05	0.32
35	18	2,?	1.5	3.6	2.03	0.52	1.0	2.2	1.56	0.35
36	18	?	2.0	2.8	2.31	0.21	0.9	3.0	2.07	0.69
37	18	2,?	2.3	2.8	2.57	0.16	1.0	3.4	2.21	0.79
38	18	?	0.1	1.7	0.58	0.41				
39	19	?	0.4	0.9	0.65	0.15	0.7	2.3	1.29	0.46
40	18	2-m	1.7	3.2	2.50	0.42	0.5	3.3	1.82	1.01
41	19 + 19	? + ?	1.0	2.1	1.40	0.32	1.5	5.7	2.87	1.18
42	19	2,?	0.1	0.6	0.26	0.09	0.2	2.0	0.93	0.55
43	19	2,?	0.4	0.6	0.49	0.07	0.4	1.1	0.70	0.20
44	19	2,?	0.5	0.8	0.65	0.09	0.5	1.8	1.04	0.35
45	18	*n*	2.0	4.9	3.31	0.81	4.6	9.4	6.62	1.24
46	20	?	0.1	0.3	0.16	0.05	0.2	2.1	0.93	0.62
47	19	?	0.3	0.6	0.44	0.11	0.4	0.9	0.60	0.13
48	20	2,?	0.1	0.3	0.19	0.05	0.0	0.8	0.13	0.19

Compound numbers correspond to the peak numbering in Fig. 2

^a^In peaks 21, 23 and 41 two unseparated alcohols were detected

^b^Location of methyl groups; 2,?-methyl group next to C2 plus other branch(es) in unknown position(s); *n*-unbranched; ?-unidentified

### Composition of preen waxes-fatty acids

In gulls captured in winter fatty acids with at least one methyl group attached to the carbon chain were the most abundant (23 out of 25 detected fatty acids) and 19 out of 25 fatty acids had a methyl group next to the second carbon atom. Only two fatty acids had no substituent in the carbon chain. Among them, *n*-octanoic acid dominated, comprising from 12 to 30% of all detected fatty acids.

In breeding individuals *n*-octanoic acid also dominated, ranging from 22 to 48% of all detected fatty acids. Almost all fatty acids had at least one branch in the carbon chain and methyl group was predominantly attached to the second carbon atom. In nine out of 17 breeding individuals no 2-monomethyl-branched fatty acids could be observed.

### Composition of preen waxes-alcohols

Due to the very high number of detected compounds, some alcohols could not be clearly separated. For this reason, peak nos. 21, 23 and 41 represent two alcohols each (see Fig. 2; Table 2). Unbranched hexadecanol was the alcohol that dominated in both wintering (13–26%) and breeding gulls (22–41% of all detected alcohols). The percentage of the second unbranched alcohol (*n*-octadecanol) was lower and ranged between 2 and 5% in winter, and between 5 and 9% in the breeding season. All other alcohols had at least one methyl group attached to the carbon chain and in most cases it was next to the second carbon atom. Contrary to 2-monomethyl-substituted fatty acids, 2-monomethyl-branched alcohols were detected in all breeding individuals.

### Comparison of preen wax compositions in winter and the breeding season

Sixteen out of 29 fatty acids and 33 out of 51 alcohols (represented by 31 peaks due to the insufficient separation of alcohols in two cases: peak no. 23 and peak no. 41) with an average abundance over 1% in at least one season were selected for the comparison.

*Fatty Acids.* The principal component analysis based on the relative composition of the fatty acid fraction resulted in two principal components which explained together 90.7% of the total variance in the dataset. The eigenvalues of both components were higher than one and were 12.30 for PC1 and 2.21 for PC2. The factor loadings for the two principal components are given in Table 3.

**Table 3:** Component loadings for 16 fatty acids detected in preen waxes of adult herring gulls captured in winter and during the breeding season

Peak no.	Total carbon atom number	Compound	PC1	PC2
3	8	*n*-octanoic acid	**−0.8798**	0.0547
5	10	2,6-dimethyloctanoic acid	**0.9674**	−0.1434
8	11	2,6-dimethylnonanoic acid	0.5845	**−0.7876**
11	12	2,6-dimethyldecanoic acid	**0.9941**	−0.0275
12	12	2,8-dimethyldecanoic acid	**0.9877**	0.0580
13	13	Unidentified	**−0.9534**	−0.2314
17	13	2,6-dimethylundecanoic acid	0.6703	−0.5592
18	13	2,8-dimethylundecanoic acid	**0.9207**	−0.1695
19	14	2,4,8-trimethylundecanoic acid	**−0.9395**	−0.2832
22	14	2,6-dimethyldodecanoic acid	**0.8021**	0.5330
23	14	2,8-dimethyldodecanoic acid	**0.7017**	0.6436
24	15	Unidentified	**−0.9570**	0.1206
25	14	Unidentified	**0.9334**	0.3412
26	15	2,6,10-trimethyldodecanoic acid	**−0.9404**	0.0657
28	15	Unidentified	**−0.7153**	0.5035
29	16	2,6,?-trimethyltridecanoic acid	**−0.9363**	−0.0014

Loadings with absolute values higher than 0.7 are marked in bold. Principal component no. 1 (PC1) explained 76.9% of the total variance and PC2 represented 13.8% of the variance. Compound numbers correspond to the peak numbering in Fig. 1

**Fig. 3:**
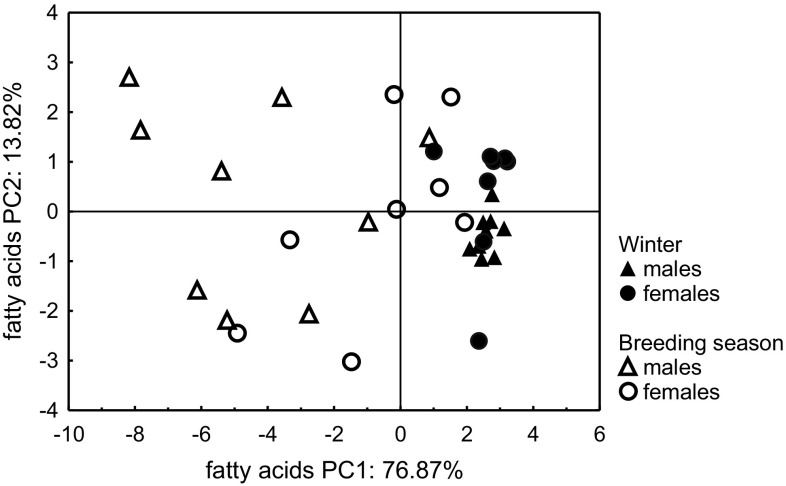
The results of principal component analysis: score plot of cases for the first two PCs based on the relative content of 16 fatty acids present in the preen waxes of adult herring gulls captured in winter and during the breeding season

**Fig. 4:**
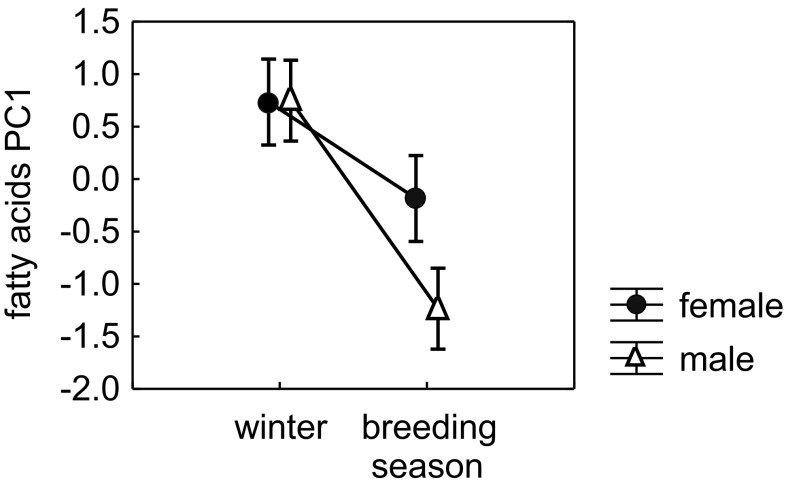
Differences in mean values of the principal component no. 1, depending on season and sex. Higher PC1 scores indicate a higher relative content of dimethyl-substituted fatty acids and a lower content of *n*-octanoic acid and trimethyl-substituted fatty acids. *Vertical lines* indicate standard error values

The separation in PC1 between herring gulls captured in winter and the breeding season could be observed (Fig. 3). However, points representing breeding gulls in the PCA score plot were widely spread (the standard deviation for males in PC1 was 0.03 in winter and 0.29 in the breeding season; for PC2 these values were: 0.09 in winter and 0.42 in the breeding season, respectively; for females in PC1 the standard deviation values were 0.07 in winter and 0.24 in the breeding season; for PC2 these values were: 0.31 in winter and 0.46 in the breeding season, respectively). Concluding, the relative contents of fatty acids in breeding gulls were very variable, contrary to wintering herring gulls, which seemed to be much more similar. The mean values of PC1 differed significantly between seasons (MANOVA, *F*_1,30_ = 55.65, *P* < 0.001) and sexes (MANOVA, *F*_1,30_ = 7.12, *P* = 0.01) with lower values of PC1 in breeding gulls than wintering gulls and lower values of PC1 in males than in females (Fig. 4). This indicates that breeding individuals (when compared to gulls from winter) as well as males (when compared to females) had a higher content of *n*-octanoic acid and trimethyl-substituted fatty acids and a lower content of dimethyl-substituted fatty acids. A significant interaction between sex and season was also observed (MANOVA, *F*_1,30_ = 7.49, *P* = 0.01), with significantly larger changes in fatty acid composition in breeding males than in breeding females (Fig. 4). The mean values of PC2 did not show a significant differentiation between the seasons (MANOVA, *F*_1,30_ = 0.08, *P* = 0.78) or the sexes (MANOVA, *F*_1,30_ = 0.15, *P* = 0.70) of adult herring gulls.

*Alcohols*. The principal component analysis based on the relative composition of the alcohol fraction resulted in three principal components that represented 88.4% of the total variance in the composition of alcohols in adult herring gulls. The eigenvalues of these components were 16.98 for PC1, 6.07 for PC2 and 4.34 for PC3. Factor loadings for the three principal components are given in Table 4.

**Table 4:** Component loadings for 33 alcohols detected in preen waxes of adult herring gulls captured in winter and in the breeding season

Peak no.	Total carbon atom number^a^	Branch^b^	PC1	PC2	PC3
4	13	2-m	**0.9317**	−0.2912	0.0467
6	14	2,?	**0.8098**	−0.5144	0.1603
8	14	2-m	**0.9576**	−0.2152	0.0848
10	15	2,?	−0.2078	**−0.7535**	0.5888
11	15	2,?	−0.4491	−0.6717	0.4630
12	15	2,?	−0.0368	**−0.8930**	0.3870
13	14	?	**−0.7961**	0.1729	−0.2362
15	15	2-m	**0.9947**	−0.0095	−0.0472
17	16	2,?	**0.8961**	0.0658	0.3652
18	16	2,?	**0.8871**	−0.1589	0.2257
19	16	2,?	0.1780	0.1910	0.6561
20	16	2,?	**0.8527**	0.4046	0.1504
22	16	2-m	**0.9699**	0.1392	0.1260
23	16 + 17	? + ?	**−0.7606**	0.2189	0.3875
24	17	2,?	−0.2540	0.0947	**0.9142**
25	17	2,?	−0.6811	0.2099	0.6406
26	17	2,?	−0.2370	−0.4009	**0.8021**
28	17	?	−0.4383	0.5739	0.5248
29	16	*n*	**−0.9142**	−0.1588	−0.2714
30	18	?	**−0.9782**	−0.0029	0.1219
31	17	2-m	**0.9815**	0.1302	−0.0904
33	18	?	**0.7487**	0.4642	0.3180
34	18	2,?	0.0436	**0.8191**	0.1967
35	18	2,?	0.6246	−0.3245	0.2296
36	18	?	0.6436	0.6889	0.2310
37	18	2,?	**0.7496**	0.6159	0.1598
39	19	?	**−0.8816**	0.3073	0.2264
40	18	2-m	**0.8957**	0.3637	−0.0515
41	19 + 19	? + ?	**−0.8578**	0.4206	0.2205
44	19	2,?	−0.4774	**0.8323**	0.1523
45	18	*n*	**−0.8944**	0.0563	−0.2755

Loadings with absolute values higher than 0.7 are marked in bold. Principal component no. 1 represented 54.8% of the total variance, PC2 explained 19.6% of the total variance and PC3 explained 14.0% of the total variance. Compound numbers correspond to the peak numbering in Fig. 2

^a^In peaks 23 and 41 two unseparated alcohols were detected

^b^Location of methyl groups; 2?-methyl group next to C2 plus other branch(es) in unknown position(s); *n*-unbranched; ?-unidentified

**Fig. 5:**
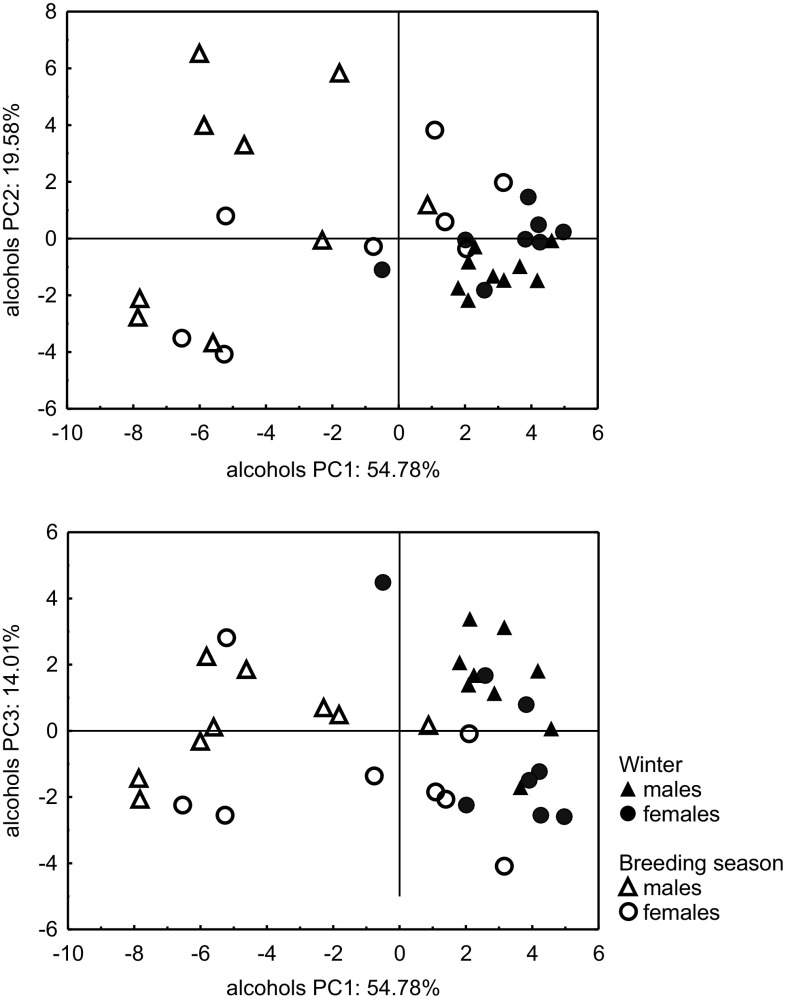
The results of principal component analysis: score plots of cases for PCs no. 1 and 2 (*upper*) and no. 1 and 3 (*lower*), based on the relative content of 33 alcohols present in the preen waxes of all adult herring gulls captured in winter and in the breeding season

The PCA score plots based on the relative composition of the alcohol fraction in the preen waxes of adult herring gulls showed results similar to the analysis based on fatty acid content. There was a clear separation between gulls (particularly males) captured in winter and in the breeding season. The dispersion of points representing breeding gulls indicates that the variation in the composition of the alcohol fraction was much higher in the waxes of breeding birds than in wintering ones (Fig. 5).

**Fig. 6:**
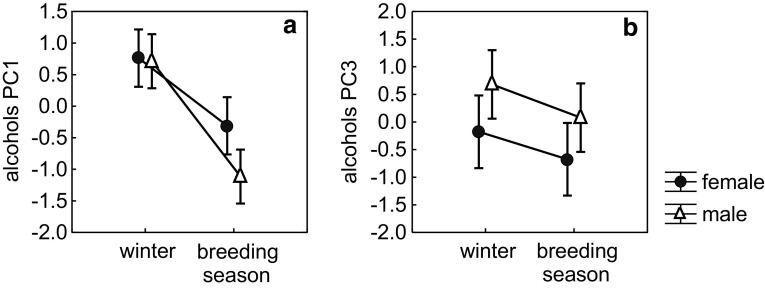
Differences in mean values of PC1 (**a**) and PC3 (**b**), depending on the season and bird sex. Higher PC1 scores indicate a higher relative content of 2-monomethyl-branched alcohols and a lower content of *n*-hexadecanol, while higher PC3 scores indicate a higher relative content of two unidentified c17 alcohols. *Vertical lines* indicate standard error values

Gulls caught in winter had significantly higher values of PC1 than breeding ones (MANOVA, *F*_1,30_ = 45.04, *P* < 0.001; Fig. 6a), which was reflected in the higher content of 2-monomethyl-substituted alcohols and the lower content of unbranched alcohols in gulls during winter. No significant difference in PC1 between sexes could be detected (MANOVA, *F*_1,30_ = 3.90, *P* = 0.06), as well as no differences being found in PC2 between sexes (MANOVA, *F*_1,30_ = 0.04, *P* = 0.84) or seasons (MANOVA, *F*_1,30_ = 2.22, *P* = 0.15). PC3 differed only between sexes (MANOVA, *F*_1,30_ = 6.62, *P* = 0.02), with higher values reached in females (Fig. 6b), indicating a lower content of two unidentified C_17_ alcohols than in males. It did not differ between seasons (MANOVA, *F*_1,30_ = 3.07, *P* = 0.09).

The effect of the interaction between sex and season on PC1 was similar to that observed in fatty acids, i.e., changes in the relative contents of selected alcohols between winter and the breeding season were more pronounced in males (Fig. 6a); however, this interaction was not significant (MANOVA, *F*_1,30_ = 3.06, *P* = 0.09). Additionally, no significant interactions between sex and season were detected in the two remaining PCs (PC2: MANOVA, *F*_1,30_ = 2.25, *P* = 0.14; PC3: MANOVA, *F*_1,30_ = 0.03, *P* = 0.87).

## Discussion

This study showed for the first time that adult herring gulls in both winter and during the breeding season produced monoester waxes, which are often major components of preen gland secretion and are commonly found in many bird species of various orders, including Charadriiformes (Jacob 1978a). Herring gulls produced high amounts of *n*-octanoic acid and *n*-hexadecanol, followed by numerous compounds with methyl branches, including 2-monomethyl-substituted fatty acids and alcohols, of lower relative contents. Very similar results were already reported by Zeman and Jacob (1972). Recent studies conducted on intact waxes collected in the pre-laying period from black-legged kittiwakes (a species related to herring gulls) have shown that two main compounds in this species were hexadecyl octanoate and octadecyl octanoate (Leclaire et al. 2012), which suggests a similar wax composition, at least to some degree.

The chemical composition of preen wax components in herring gulls varied between winter and the breeding season with much higher contents of unbranched compounds and lower contents of dimethyl-substituted fatty acids during the breeding season. The differences observed between breeding and winter birds did not seem to be influenced by the year-effect, as the results obtained for two breeding individuals captured in 2011 did not differ from the results for other breeding individuals captured in 2012. Winter and breeding gulls were captured in different locations with a distance of more than 80 km between them. Factors like different seasons and locations could result in different diets, which have been shown to be one of the factors affecting the chemical composition of preen waxes (Thomas et al. 2010). All gulls are omnivorous and opportunistic, feeding mainly on fish, marine invertebrates or offal and refuse (Cramp and Simmons 1983; Burger and Gochfeld 1996). Lipids produced by marine organisms are usually unsaturated and composed of C_16_ and longer fatty acids (Nevenzel 1970). Fatty acid components of preen waxes in herring gulls had the main chain not longer than C_16_ and were all saturated, thus it seems unlikely that diet has a decisive influence on the variability of the chemical composition of preen waxes in this species.

A switch from mono- to diester waxes could be observed in red knots and in 18 other sandpiper species during incubation (Reneerkens et al. 2002). As suggested by Reneerkens et al. (2006), this shift may lead to changes in the physical properties of waxes to reduce the smell of birds and diminish the risk of predation, reduce feather abrasion, or even inhibit the growth of pathogens (Reneerkens et al. 2008). The change in the chemical composition of waxes is under endogenous control, but depends also on external factors, like environmental conditions and food availability (Reneerkens et al. 2007b). Gulls, as well as sandpipers, belong to the order Charadriiformes and, as there were suggestions that the chemical composition of preen gland secretion can be used as a chemotaxonomic tool (Jacob 1978, but see Levy and Strain 1982), some similarities in the preen wax composition between red knots and herring gulls could be expected. However, diester waxes were not found in the preen gland secretion of breeding gulls. Red knots breed separately on the ground with their nests hidden in low vegetation (van Gils and Wiersma 1996), thus they are exposed to mammalian predators, like arctic foxes (*Vulpes lagopus*) which use smell to detect their prey. In contrast, gulls breed in colonies which are visible from afar and usually have a strong odour caused by the abundance of faecal matter. In a colony, the risk of mammalian predation is diluted and birds can warn each other against danger and collectively defend the colony (Götemark and Andersson 1985). Additionally, breeding colonies of herring gulls captured during this study were located on the roofs of buildings, which may result in limited access to the nests. For these reasons, producing and applying diesters which are more viscous than monoesters thus probably requiring higher energy costs (Reneerkens et al. 2005) is not needed so much. On the other hand, defending the colony is a response to diurnal predators, whereas a response to nocturnal predation is usually fleeing behaviour or a lack of any response (Southern and Southern 1979; Southern et al. 1982), so some nocturnal anti-predator adaptation would be beneficial. Especially that some species of the Mustelidae family, as well as feral cats, could be seen in the breeding colony in Łeba preying on eggs and chicks (M. Knitter, personal observation).

However, there were some changes in preen wax composition observed between winter and the breeding season. Preen waxes of breeding gulls were composed of a higher relative content of *n*-octanoic acid and *n*-hexadecanol, which in some individuals was several times higher than other (branched) compounds. It is known that saturated, linear fatty acids and alcohols (as well as other organic compounds) have a higher melting temperature and are more viscous than multi-branched substances with the same molecular weight (Gibbs 2002; Knothe 2005; Rodrigues et al. 2006). Thus, although no diesters could be found in herring gulls, changes in preen gland secretion during the breeding season may have a similar function, increasing clutch protection and plumage maintenance, but to less an extent than diesters and with a much lower metabolic cost.

Changes in the chemical composition of preen gland secretion observed between winter and the breeding season, e.g., a higher content of *n*-octanoic acid and *n*-hexadecanol and a lower content of 2,6- and 2,8-dimethyl-substituted fatty acids were significantly larger in breeding males than breeding females. In herring gulls, the males and females share incubation duties (Drent 1970; Morris 1987). It has been observed that each partner incubates more often at a particular time of the day, but a tendency that one particular sex incubates more often in one period of the day, e.g., males at night and females during the day, has not been reported before (Morris 1987). However, in the breeding colony in Łeba, where samples of preen waxes were collected, males were observed to incubate more often at night and females during the day (M. Knitter, unpublished data). Gulls breed in colonies and anti-predator warning is a typical behaviour observed there, but only during the daytime (Burger and Gochfeld 1996). Thus, chemical changes in preen wax composition in males may evolve as additional nocturnal protection against mammalian predators which use olfaction to detect their prey and which are more active at night.

It is widely accepted that it is possible not only to differentiate between species, but also between sexes and individuals based exclusively on the composition of uropygial secretion (Bonadonna and Nevitt 2004; Bonadonna et al. 2007; Reneerkens et al. 2007a; Mardon et al. 2010). Hence, it cannot be excluded that these chemical changes could improve or alter the visual characteristics of the plumage or they could also alter the odour of the birds, making potential mates or specific individuals easier to identify, which was found in other birds (Hagelin et al. 2003; López-Rull et al. 2010; Pérez-Rodríguez et al. 2011).

A further study (Mardon et al. 2011) proved that the majority of lipids present on the plumage of blue petrels originated from uropygial secretion, suggesting a major role of preen waxes in mate recognition. Moreover, the presence of sex and individual odour signatures, based on wax esters of similar structures, was also reported for the herring gull-related black-legged kittiwake (Leclaire et al. 2011). Therefore, while it is impossible to judge the functional role of the preen waxes of herring gulls and changes in their composition over the course of time without further experiments, protection against mammalian predators and olfactory mate recognition could both be suggested based on the results of the current study.
